# Supplementary material for: Purification Process and In Vitro and In Vivo Bioactivity Evaluation of Pectolinarin and Linarin from Cirsium japonicum
Source: Molecules. 2022 Dec 8;27(24):8695. doi: 10.3390/molecules27248695 (PMC9780979; doi:10.3390/molecules27248695)
Supplement: Supplementary file 1 [file molecules-27-08695-s001.zip › molecules-2038155-supplementary.pdf]

Supplementary material

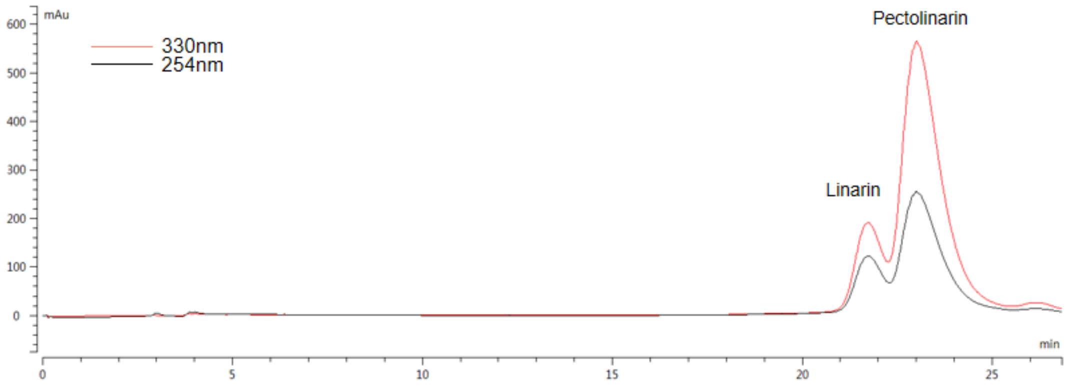

Figure S1. Pre-HPLC chromatograms of pectolinarin and linarin.

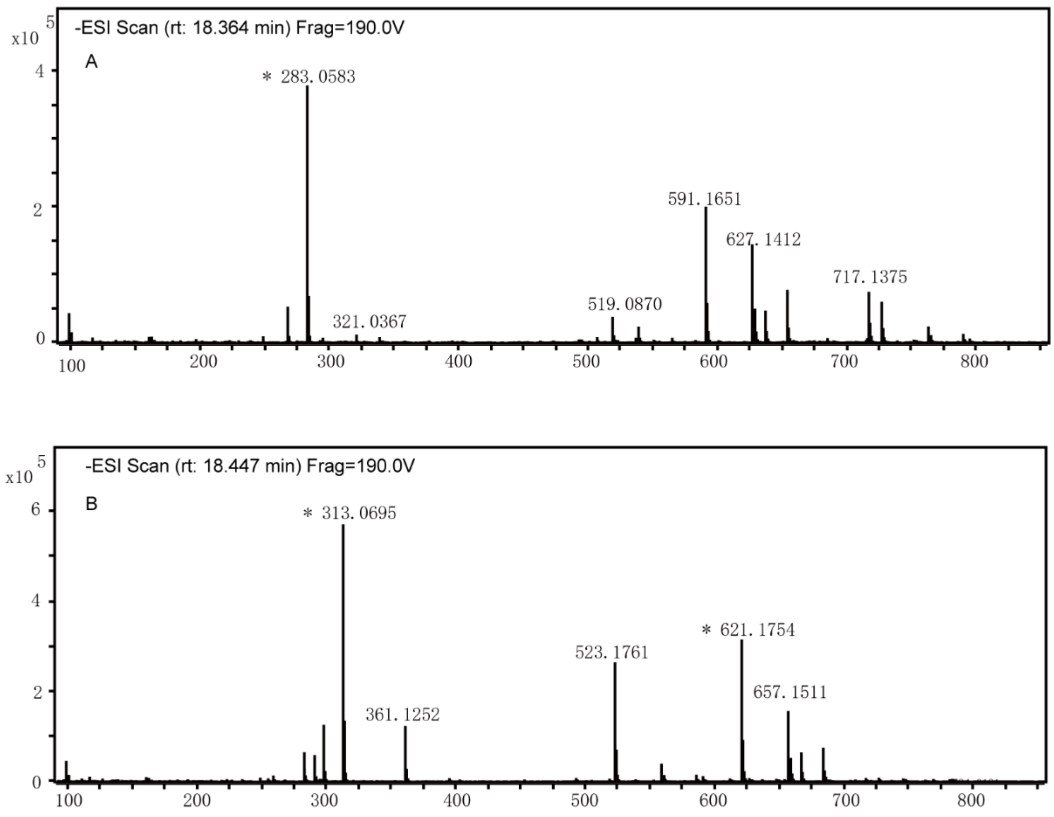

Figure S2. HR-ESIMS spectrum of linarin (A) and pectolinarin (B). * represents the peak with the highest or relatively high response.

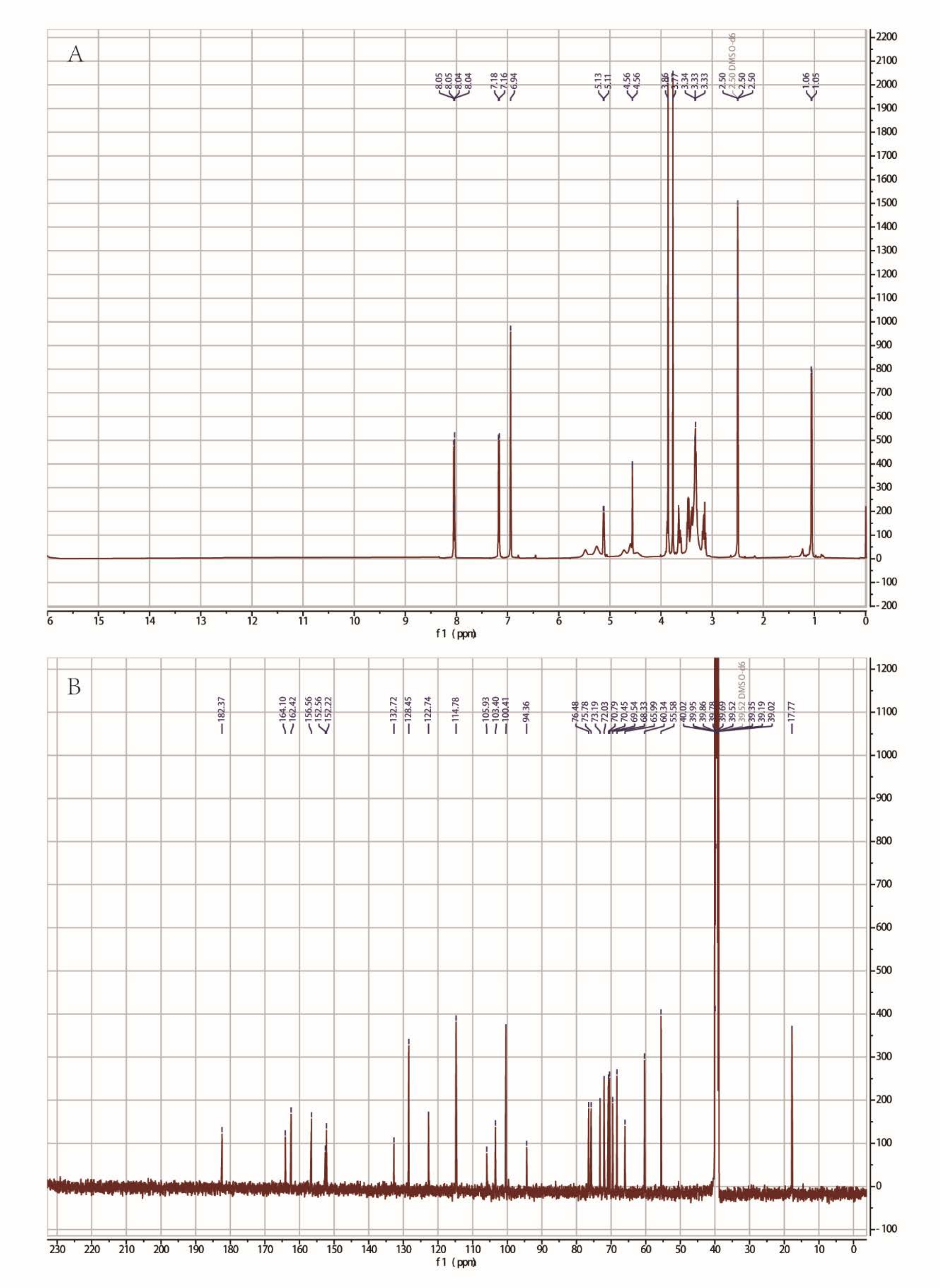

Figure S3. ^{1}H- NMR (A) and ^{13}C- NMR (B) spectrum of pectolinarin.

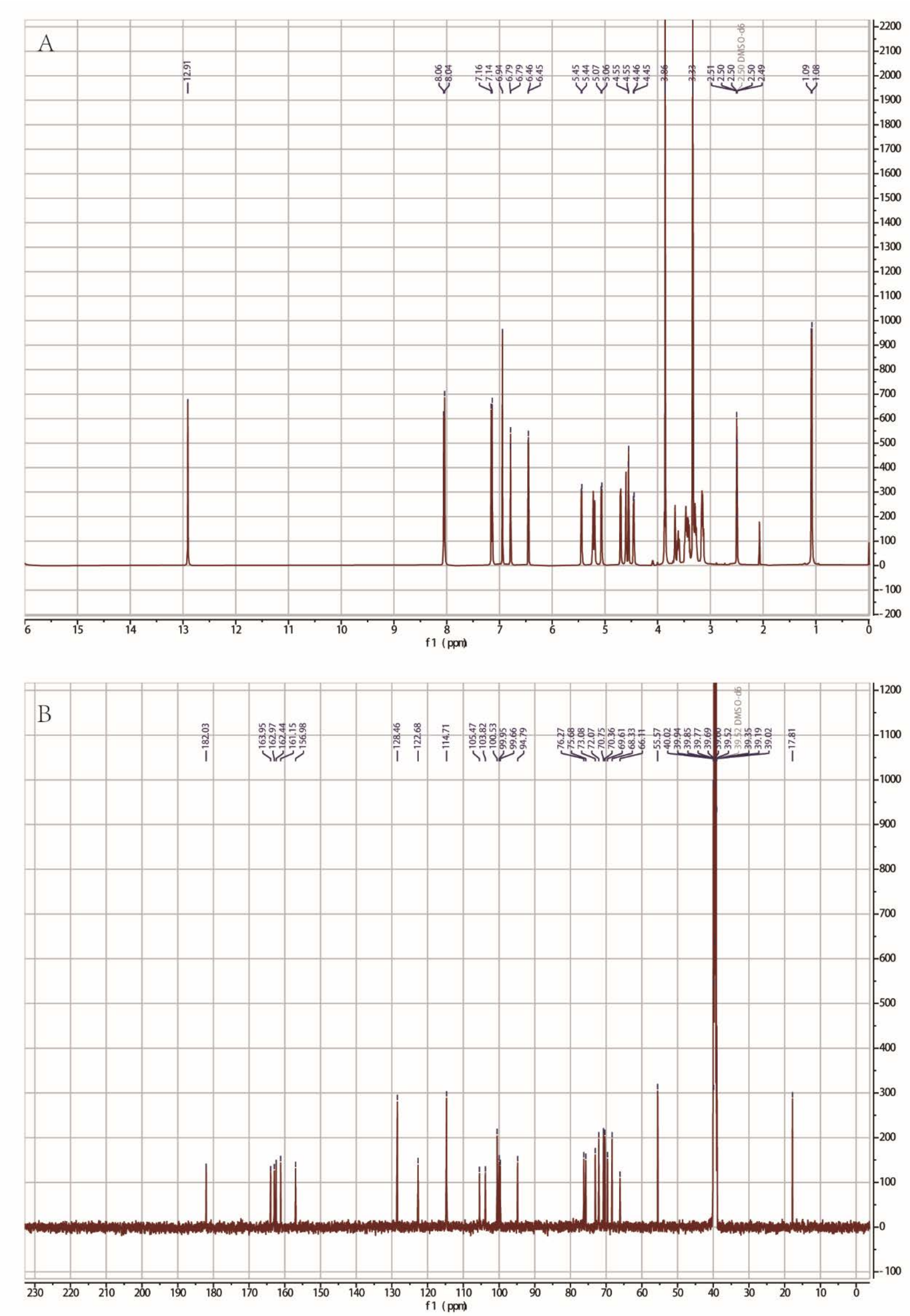

Figure S4. ^{1}H- NMR (A) and ^{13}C- NMR (B) spectrum of linarin.

Method S1: Non-targeted metabolomics analysis

Experimental non-targeted metabolomics analysis of mice serum was performed as previously described [4]. Briefly, 20 µL of serum was mixed with 10 µL of nonadecanoic acid methanol solution (1 mg/mL, w/v) and 250 µL of H_2O-MeOH-$CHCl_3$ solution (2:5:2, v/v/v). Then, the resulted mixture was kept at 4℃ for 20 min. Next, the mixture centrifuged at 14,000 rpm for 15 min at 4℃. Subsequently, 200 µL of supernatant was dried in nitrogen. The residue was derivatized with 80 µL of methoxyamine pyridine solution (15 mg/mL, w/v) and incubated at 37 °C for 90 min. Then, 80 µL of bis-(trimethylsilyl) trifluoroacetamide with 1% chlorotrimethyl-silane was added and incubated at 70°C for 1 h. Nonadecanoic acid was used as internal standard substance.

Finally, the metabolic profiling was analyzed as previously reported on 7890B-5977B GC-MS (Agilent, MA, USA) with a HP-5MS column (60 m × 0.25 mm × 0.25 µm, Agilent, MA, USA). The temperature was programmed to 60°C, 1 min → 60–100°C (8°C/min), 5 min → 100–170°C (15°C/min), 5 min → 170–210°C (10°C/min), 5 min → 210–350°C (10°C/min), 5 min. The auxiliary temperature was 240°C. The injector temperature was 250°C and injection volume was 1.0 µL. The helium carrier gas flow rate was set at 1 mL/min. MS detection was used selective ion scanning m/z 50–600 amu. The EI source was 230°C. The MS quadrupole were maintained at 150°C.

The data analysis was performed as previously described [1]. In brief, all the GC-MS raw data were subjected to batch molecular feature extraction by using MassHunter Profinder_B.08 (Agilent Co., Ltd, CA, USA). Then, the generated data were exported to Excel (Microsoft, Redmond, WA, USA) and used in the subsequent multivariate analysis. All the raw data were stored at Guangdong Metabolic Disease Research Centre of Integrated Medicine, which will be available upon request. Unsupervised PCA and supervised OPLS-DA analysis were performed on SIMCA-P 13.0 software (Umetrics, Umeå, Sweden) to identify plasma metabolites contributing to the differences between the two groups. All variables were Pareto-scaled prior to analyses. Here, VIP (Variable Importance in Projection) >1.0 and $p < 0.05$ were set as a statistical threshold for discriminating key differential metabolites.

[1] Li, K., He, Z., Wang, X., Pineda, M., *et al.* Apigenin C-glycosides of *Microcos paniculata* protects lipopolysaccharide induced apoptosis and inflammation in acute lung injury through TLR4 signaling pathway. Free Radic Biol Med 2018, 124, 163-175. https://doi.org/10.1016/j.freeradbiomed.2018.06.009.
